# Protective Effects of Sophoraflavanone G by Inhibiting TNF-α-Induced MMP-9-Mediated Events in Brain Microvascular Endothelial Cells

**DOI:** 10.3390/ijms25010283

**Published:** 2023-12-24

**Authors:** Tsong-Hai Lee, Jiun-Liang Chen, Ming-Ming Tsai, Yi-Hsuan Wu, Hui-Ching Tseng, Li-Ching Cheng, Velayuthaprabhu Shanmugam, Hsi-Lung Hsieh

**Affiliations:** 1 Stroke Center and Stroke Section, Department of Neurology, Chang Gung Memorial Hospital, and College of Medicine, Chang Gung University, Taoyuan 333, Taiwan; thlee@cgmh.org.tw; 2 Division of Chinese Internal Medicine, Center for Traditional Chinese Medicine, Chang Gung Memorial Hospital, and School of Traditional Chinese Medicine, College of Medicine, Chang Gung University, Taoyuan 333, Taiwan; a12015@cgmh.org.tw; 3 Division of Basic Medical Sciences, Department of Nursing, Research Center for Chinese Herbal Medicine, and Graduate Institute of Health Industry Technology, Chang Gung University of Science and Technology, Taoyuan 333, Taiwan; mmtsai@mail.cgust.edu.tw (M.-M.T.); yhwu03@mail.cgust.edu.tw (Y.-H.W.); hctseng@mail.cgust.edu.tw (H.-C.T.); victoria@mail.cgust.edu.tw (L.-C.C.); 4 Department of General Surgery, New Taipei Municipal Tucheng Hospital, New Taipei 236, Taiwan; 5 Department of General Surgery, Chang Gung Memorial Hospital, Taoyuan 333, Taiwan; 6 Department of Biotechnology, Bharathiar University, Coimbatore 641046, India; velayuthaprabhu@buc.edu.in; 7 Department of Neurology, Chang Gung Memorial Hospital, Taoyuan 333, Taiwan

**Keywords:** sophoraflavanone G, brain microvascular endothelial cells, matrix metalloproteinase-9, zonula occludens-1, brain protection

## Abstract

The regulation of matrix metalloproteinases (MMPs), especially MMP-9, has a critical role in both physiological and pathological events in the central nervous system (CNS). MMP-9 is an indicator of inflammation that triggers several CNS disorders, including neurodegeneration. Tumor necrosis factor-α (TNF-α) has the ability to stimulate the production of different inflammatory factors, including MMP-9, in several conditions. Numerous phytochemicals are hypothesized to mitigate inflammation, including the CNS. Among them, a flavonoid compound, sophoraflavanone G (SG), found in *Sophora flavescens* has been found to possess several medicinal properties, including anti-bacterial and anti-inflammatory effects. In this study, mouse brain microvascular endothelial cells (bMECs) were used to explore TNF-α-induced MMP-9 signaling. The effects of SG on TNF-α-induced MMP-9 expression and its mechanisms were further evaluated. Our study revealed that the expression of MMP-9 in bMECs was stimulated by TNF-α through the activation of ERK1/2, p38 MAPK, and JNK1/2 via the TNF receptor (TNFR) with a connection to the NF-κB signaling pathway. Moreover, we found that SG can interact with the TNFR. The upregulation of MMP-9 by TNF-α may lead to the disruption of zonula occludens-1 (ZO-1), which can be mitigated by SG administration. These findings provide evidence that SG may possess neuroprotective properties by inhibiting the signaling pathways associated with TNFR-mediated MMP-9 expression and the subsequent disruption of tight junctions in brain microvascular endothelial cells.

## 1. Introduction

Cerebrovascular cells, brain microvascular endothelial cells (bMECs), are essential components in the central nervous system (CNS). They have a crucial purpose in preserving the brain’s microenvironment and facilitating various processes such as cerebral blood flow regulation, microvascular tone modulation, and maintenance of the integrity of the blood–brain barrier (BBB) [1,2]. Nevertheless, the impairment of the vascular endothelium is an initial clinical observation, which is strongly associated with several vascular and cerebral disorders in individuals affected by atherosclerosis, stroke, and neurodegenerative conditions [3,4,5]. The disruption and dysfunction of brain microvessels can lead to subsequent damage to neuronal cells within the surrounding microenvironment [3,5]. Therefore, maintaining the integrity of the BBB to ameliorate brain disorders is a pivotal aspect in the treatment of several cerebral ailments. The potential for therapeutic intervention exists in preventing or reducing BBB disruption [6,7]. Moreover, offering protective strategies for cerebrovascular cells has received increased attention in CNS diseases.

Matrix metalloproteinases (MMPs) are a large family of zinc-dependent endopeptidases with crucial functions for their pathophysiological roles, especially in extracellular matrix (ECM) turnover [8,9]. Among those MMPs, MMP-9 plays an important role in morphogenesis, wound healing, and neurite outgrowth in the CNS [9,10]. Overexpression of MMP-9 may have a role in the pathogenesis of brain illnesses resulting from various brain traumas [10,11]. Previous studies have demonstrated that inflammatory cytokines and endotoxin could induce MMP-9 expression with their associated activity in brain cells [10,12]. MMP-9 can also potentially play a role in the BBB integrity, leading to the onset of neuroinflammation through the influence of several detrimental elements, such as lipopolysaccharides [13]. Previous reports have demonstrated that proinflammatory cytokines can induce MMP-9 expression and elicit functional alterations in brain cells, including human cerebral microvascular endothelial cells [14] and rat astrocytes [15,16]. These results suggest that proinflammatory cytokines and MMP-9 may play important roles in neural inflammation and brain diseases, arousing our interest in investigating the potential effects of phytochemical compounds on MMP-9-associated events in bMECs. Here, we aim to elucidate the potential of tumor necrosis factor-α (TNF-α), a pivotal proinflammatory cytokine, to promote the MMP-9 expression in bMECs and its implications and underlying mechanism.

Phytochemicals present in natural products have shown potential roles in reducing the risk of several disorders, especially in neurodegenerative diseases [17,18,19]. The probable underlying mechanisms may involve the inhibition of oxidative stress and the suppression of the synthesis of different proinflammatory mediators. To date, hundreds of natural products have been widely screened for anti-oxidative and anti-inflammatory properties in in vitro and in vivo experiments. Several natural products can be regarded as potential sources of highly effective molecules with antioxidant and anti-inflammatory properties, explaining their therapeutic and preventive benefits [18,19]. Flavonoids belong to natural polyphenolic compounds exerting beneficial effects in cardiovascular and neurological disorders [20]. Among these, sophoraflavanone G (SG), a flavonoid compound, has been isolated from *Sophora flavescens*, which possesses several medicinal properties, including anti-bacterial and anti-inflammatory effects [21,22]. Previous reports have indicated that some natural flavonoid compounds exhibit potential neuroprotective agents to treat neurodegenerative disorders [20,23]. Although there is extensive research reports that exist to demonstrate the effects of natural flavonoid compounds in various disorders, including brain disorders, there are no studies indicating the effects and mechanisms of SG in the CNS. Hence, we established the paradigm of tumor necrosis factor-alpha (TNF-α)-induced MMP-9 in bMECs to assess the impact of SG on MMP-9-mediated processes, specifically pertaining to the impairment of tight junction integrity.

Drawing on the aforementioned backgrounds and our prior investigations into the mechanisms of brain inflammatory responses triggered by MMP-9 induction [12], the research was conducted to evaluate the effects of SG on TNF-α-induced MMP-9 expression in bMECs. Herein, SG ameliorates the disruption of zonula occludens-1 (ZO-1) arranged integrity by TNF-α-induced MMP-9. Moreover, TNF-α-stimulated activation of TNFR-dependent MAPKs and the NF-κB signaling pathway also been inhibited by SG pretreatment. These findings indicate that the SG may possess neuroprotective properties through its anti-inflammatory properties, which help to prevent the disruption of ZO-1 organized integrity mediated by MMP-9.

## 2. Results

### 2.1. TNF-α Induces MMP-9 Expression and Its Effect on Brain Microvascular Endothelial Cells (bMECs)

**Figure 1:**
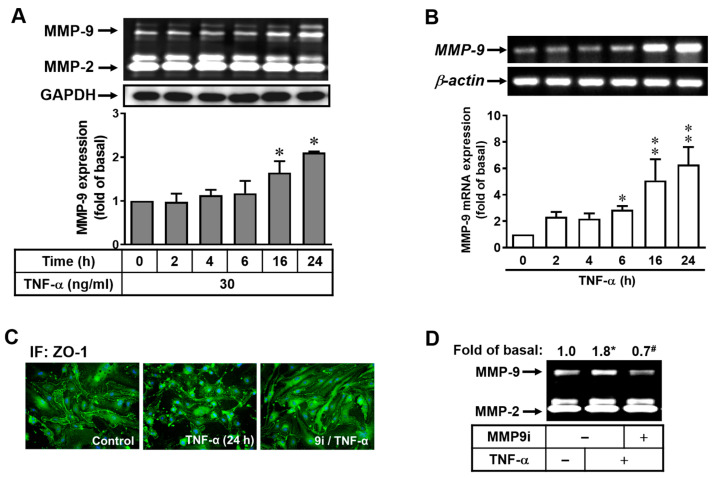
TNF-α induces MMP-9 expression and its effect on brain microvascular endothelial cells (bMECs). (**A**,**B**) TNF-α increases the expression of MMP-9 in a time-dependent manner. After treating with TNF-α (30 ng/mL) in bEnd.3 cells for the indicated time intervals, the MMP-9 expression and activity were analyzed by gelatin zymography, Western blot, and RT-PCR (GAPDH and *β-actin* as the internal controls). The intensity of zymographic and gene expression represent relative fold differences of protein and gene levels on the basis of densitometer quantitation. (**C**,**D**) MMP-9 is involved in TNF-α-induced disruption of ZO-1 arranged integrity. After growing on coverslips for 24 h, bEnd.3 cells were pretreated with MMP-9 inhibitor (9i, 1 μM) for 1 h and then incubated with TNF-α (30 ng/mL) for 24 h. After treatment, the conditioned media were collected for gelatin zymography (**D**), and the cells were fixed for immunofluorescence staining (**C**, ZO-1). The intensity of the zymographic represents relative fold differences of protein levels on the basis of densitometer quantitation. Data are shown as the mean ± SEM (*n* = 3). * *p* < 0.05; ** *p* < 0.01, as compared to untreated control. ^#^ *p* < 0.05, as compared to TNF-α treatment only. The image represents one of three individual experiments.

TNF-α is a common proinflammatory cytokine that contributes to various inflammation-related disorders, including CNS [24]. To determine whether TNF-α treatment in bMECs cells could induce MMP-9 expression, bEnd.3 cells were treated with TNF-α (30 ng/mL), and then the conditioned media were collected for gelatin zymography analysis. In Figure 1A, TNF-α-treated bMECs at the concentration of 30 ng/mL showed significant increase in MMP-9 expression in a time-dependent manner, especially between the 16 h and 24 h time points, where MMP-2 and GAPDH were used as internal controls. In order to look into the potential relationship between the transcriptional and translational expression of MMP-9 induced by TNF-α, a RT-PCR analysis was carried out. As shown in Figure 1B, the expression of MMP-9 mRNA was increased in a time-dependent manner upon TNF-α stimulation with a significant upregulation from the time point of 6 h to 24 h, where the expression of β-actin mRNA had no significant change and served as the internal control. These data revealed that the upregulation of MMP-9 by TNF-α treatment in bMECs is mediated through the MMP-9 transcriptional event.

The upregulation of MMP-9 has been demonstrated with a main contribution to several brain injuries and various CNS disorders [14]. Therefore, the effects of TNF-α-induced MMP-9 expression on the functions of bMECs for BBB formation were further investigated, specifically focusing on the maintenance of tight junction protein ZO-1 in its arrangement and integrity. As shown in Figure 1C, the images demonstrated that ZO-1 was completely associated with the plasma membrane (control); however, this integrity was disrupted following TNF-α stimulation (30 ng/mL) for 24 h (Figure 1C, middle). Moreover, the pretreatment of the MMP-9 inhibitor (9i, 1 μM) effectively suppressed the detrimental effects of TNF-α on the structural integrity of ZO-1 (9i/TNF-α), indicating that MMP-9 was indeed involved in this phenomenon. After gelatin zymography analysis to check MMP-9 expression in this particular scenario, TNF-α-induced MMP-9 expression and activity were markedly attenuated upon pretreatment of MMP-9 inhibitor (MMP9i, 1 μM; Figure 1D). These data suggested that TNF-α induces the disruption of ZO-1 arranged integrity in bMECs via the MMP-9 pathway.

### 2.2. TNF-α Induces MMP-9 Expression via the TNF Receptor-Dependent Pathway

**Figure 2:**
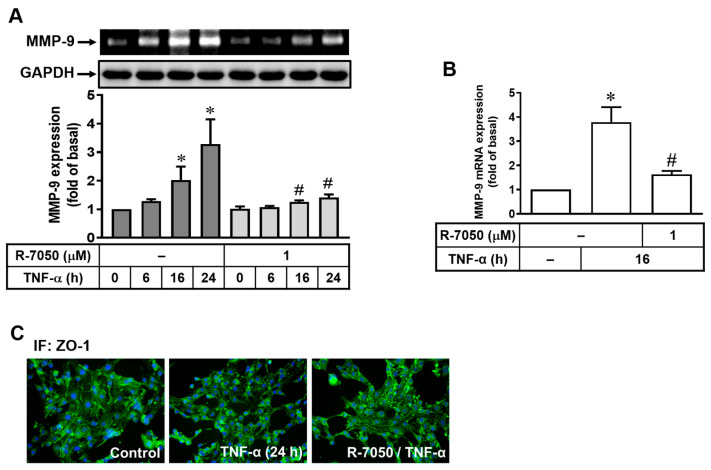
TNF-α induces MMP-9 expression via TNF receptor-dependent pathway. (**A**,**B**) Cells were pretreated with a TNF receptor antagonist R-7050 (1 μM) following TNF-α (30 ng/mL) incubation for the indicated times. After the stimulation, the MMP-9 expression and activity were analyzed by gelatin zymography, Western blot, and RT-PCR (GAPDH and *β-actin* as the internal controls). The intensity of the zymographic quantitated by densitometry and gene expression was expressed as fold of untreated control. Data are expressed as the mean ± SEM (*n* = 3). * *p* < 0.05, ^#^ *p* < 0.05, as compared to untreated control and TNF-α treatment only, respectively. (**C**) After stimulation, the cells were fixed and labeled with anti-ZO-1 antibody and a FITC-conjugated secondary antibody, and then the cell images were observed by immunofluorescence stain (ZO-1). The image represents one of three individual experiments.

In the immune system, TNF-α needs to bind to TNF receptors (TNFRs) to initiate the inflammatory reactions for effective host defenses against pathogen infection [25]. Here, to explore whether TNFR is involved in TNF-α-induced MMP-9 expression in bMECs, cells were pretreated with a TNFR antagonist R-7050 (1 μM) for 1 h and subsequently incubated with TNF-α (30 ng/mL). As shown in Figure 2A, pretreatment with R-7050 significantly blocked TNF-α-induced MMP-9 expression, demonstrating the involvement of TNFR in this specific response. Similarly, the RT-PCR data showed that pretreatment with R-7050 significantly inhibited TNF-α-induced MMP-9 mRNA expression (Figure 2B). We further confirmed the effect of TNFR in TNF-α-induced MMP-9-mediated disruption of ZO-1 arranged integrity. In Figure 2C, R-7050 pretreatment (1 μM) significantly attenuated TNF-α-induced disruption of ZO-1 arranged integrity (R-7050/TNF-α) by immunofluorescence stain. These data indicated that TNF-α-induced MMP-9-dependent disruption of ZO-1 arranged integrity is mediated through a TNFR-dependent manner in bMECs.

### 2.3. The MAPKs, Including ERK1/2, p38, and JNK1/2, Are Involved in TNF-α-Induced MMP-9 Expression

**Figure 3:**
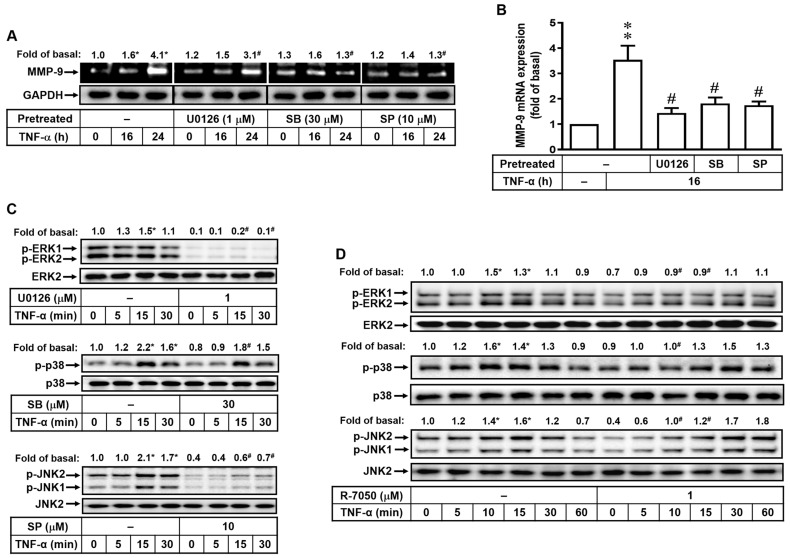
The MAPKs ERK1/2, p38, and JNK1/2 are involved in TNF-α-induced MMP-9 expression. (**A**–**C**) After pretreatment with U0126 (1 μM), SB202190 (SB, 30 μM), or SP600125 (SP, 10 μM) for 1 h, cells were stimulated with TNF-α (30 ng/mL) for different time intervals. (**A**) The conditioned media were collected and assayed for MMP-9 expression by gelatin zymography. The cell lysates were collected and analyzed with GAPDH by Western blot. (**B**) The total RNA was collected and analyzed by RT-PCR. The intensity of PCR product bands was quantitated by densitometry and expressed as fold of untreated control. Data are expressed as the mean ± SEM (*n* = 3). (**C**) The cell lysates were collected, and phosphorylation of ERK1/2 (p-ERK1/2), p38 MAPK (p-p38), JNK1/2 (p-JNK1/2), and their total proteins (ERK2, p38, and JNK2) was analyzed by Western blot. (**D**) After R-7050 pretreatment (1 μM) for 1 h and then TNF-α (30 ng/mL) stimulation, the cell lysates were collected for Western blot analysis, including ERK1/2 (p-ERK1/2), p38 MAPK (p-p38), JNK1/2 (p-JNK1/2), and their total proteins (ERK2, p38, and JNK2). * *p* < 0.05; ** *p* < 0.01, ^#^ *p* < 0.05, as compared to untreated control and the TNF-α treatment only, respectively, which indicate statistical significance. The image data represent one of three individual experiments.

Activation of MAPKs is involved in various stimuli-mediated cellular functions in brain cells [26] and MMP-9 upregulation in brain astrocytes [15]. In order to understand the involvement of MAPKs in TNF-α-induced MMP-9 expression, we used respective specific inhibitors of MAPKs, including U0126 (1 μM), SB202190 (SB, 30 μM), and SP600125 (SP, 10 μM) to pretreat bMECs for 1 h followed by TNF-α (30 ng/mL) stimulation. In Figure 3A, gelatin zymography analysis demonstrated that TNF-α-induced MMP-9 expression could be attenuated by the pretreatment of U0126, SB202190, or SP600125. To further examine the effects of MAPKs on the MMP-9 transcription event, the RT-PCR analysis was performed, and results showed that U0126, SB202190, or SP600125 pretreatment inhibited TNF-α-induced MMP-9 mRNA expression (Figure 3B). All these data indicated that MAPKs, including ERK1/2, p38, and JNK1/2, are involved in TNF-α-induced MMP-9 expression in bMECs. Moreover, TNF-α stimulated the phosphorylation of MAPKs in a time-dependent manner with a maximal response within 15 min and sustained over 30 min (Figure 3C), which could also be inhibited by pretreatment with respective MAPK specific inhibitors. The findings of this study indicated that the upregulation of MMP-9 by TNF-α in bMECs is facilitated through the activation of ERK1/2, p38, and JNK1/2 signaling pathways. Furthermore, the cells were pretreated with R-7050 (1 μM) to ascertain if the phosphorylation of MAPKs induced by TNF-α is facilitated through the TNFR-dependent pathway. R-7050 pretreatment significantly inhibited TNF-α-stimulated phosphorylation of MAPKs (Figure 3D), demonstrating that TNF-α stimulated the phosphorylation of ERK1/2, p38, and JNK1/2 through a TNFR-mediated pathway in these cells.

### 2.4. Role of NF-κB in TNF-α-Induced MMP-9 Expression in bMECs

**Figure 4:**
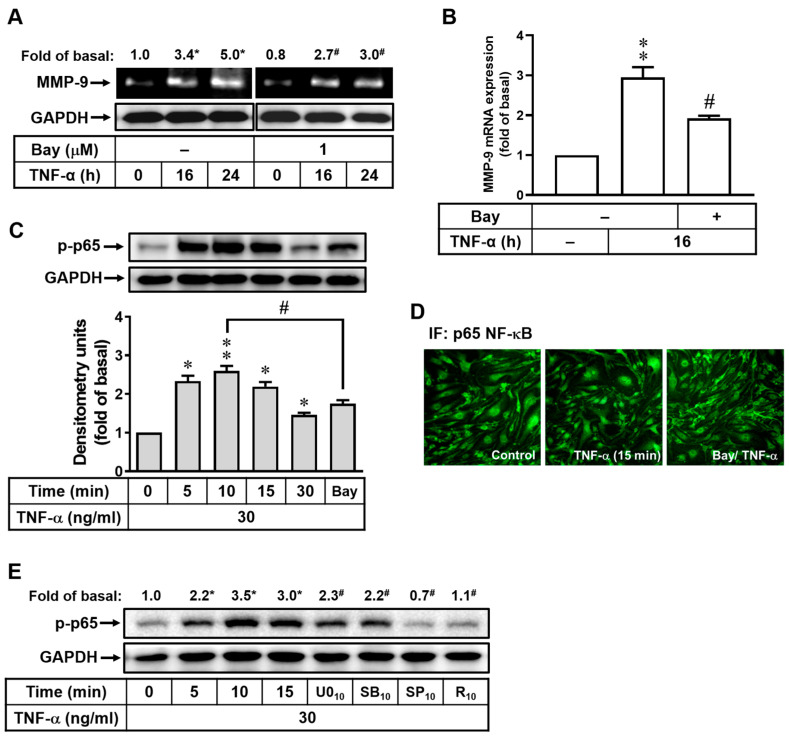
The participation of NF-κB in TNF-α-induced MMP-9 expression in bMECs. (**A**,**B**) After pretreatment with Bay11-7082 (Bay, 1 μM) for 1 h, cells were stimulated with TNF-α (30 ng/mL) for the indicated time intervals. Conditioned media and total RNA were collected for MMP-9 activity and gene expression, respectively, using gelatin zymography and RT-PCR. (**C**) TNF-α increases phosphorylation of p65 NF-κB in cells treated with TNF-α (30 ng/mL) in a time-dependent manner, and it was attenuated in cells pretreated with Bay (1 μM) for 1 h followed by TNF-α treatment for 10 min. (**D**) Representative immunofluorescence images from cells pretreated with Bay (1 μM) for 1 h, TNF-α (30 ng/mL) stimulation for 15 min, and then fixed and labeled with anti-NF-κB p65 antibody/FITC-conjugated secondary antibody. (**E**) Expression pattern of p-p65 in cells pretreated with U0126 (U0, 1 μM), SB202190 (SB, 30 μM), SP600125 (SP, 10 μM), and R-7050 (R, 1 μM) for 1 h and TNF-α (30 ng/mL) for the indicated times. The cell lysates were collected for Western blot analysis to determine phosphorylation of p65 NF-κB (p-p65) (**C**,**E**) and GAPDH (**A**,**C**,**E**). Data are expressed as the mean ± SEM (*n* = 3). * *p* < 0.05; ** *p* < 0.01, as compared to the untreated control. ^#^ *p* < 0.05, as compared to the cells stimulated with TNF-α only. The image represents one of three individual experiments.

The involvement of NF-κB-dependent pathways in MMP-9 expression has been documented in various cell types [12]. In order to determine more dynamically whether the TNF-α-induced MMP-9 expression is mediated through NF-κB activation in bMECs, a NF-κB inhibitor (Bay11-7082) was used. Pretreatment with Bay11-7082 (Bay, 1 μM) in bMECs significantly inhibited TNF-α-induced MMP-9 expression (Figure 4A), suggesting that NF-κB was involved in this event. Furthermore, the results of RT-PCR analysis demonstrated that Bay pretreatment attenuated TNF-α-induced MMP-9 mRNA expression (Figure 4B). These results indicated that the transcription factor NF-κB participates in the signaling of TNF-α-induced MMP-9 expression in bMECs. Furthermore, TNF-α stimulated time-dependent phosphorylation of p65 (a NF-κB subunit) in these cells (Figure 4C) with a significant increase within a time frame of 5 to 30 min, where the event was markedly attenuated by Bay pretreatment (1 μM) (Figure 4C). We further confirmed the TNF-α-stimulated NF-κB activation by immunofluorescence stain with a p65 subunit antibody to assess the nuclear translocation of p65 NF-κB. As shown in Figure 4D, TNF-α-stimulated translocation of p65 from the cytosol into the nucleus. On the other hand, pretreatment with Bay (1 μM) inhibited TNF-α-stimulated translocation of p65 (Figure 4D). These data indicated that activation of NF-κB, including phosphorylation and translocation of the p65 subunit, is critical for TNF-α-induced MMP-9 expression in bMECs. Moreover, as shown in Figure 4E, pretreatment with U0126 (U0, 1 μM), SB202190 (SB, 30 μM), or R-7050 (R, 1 μM) significantly inhibited TNF-α-stimulated p65 NF-κB phosphorylation at 10 min, suggesting that TNF-α stimulated p65 NF-κB activation (phosphorylation) via TNFR-dependent MAPKs pathways in bMECs. According to the obtained results, we demonstrated that TNF-α induces MMP-9 expression via TNFR-dependent activation of ERK1/2, p38, and JNK1/2 linking to NF-κB cascade in bMECs.

### 2.5. Sophoraflavanone G, a Natural Flavanone Compound, Inhibits TNF-α-Induced MMP-9 Expression by Attenuating TNFR/MAPK (ERK1/2)/NF-κB Cascade

**Figure 5:**
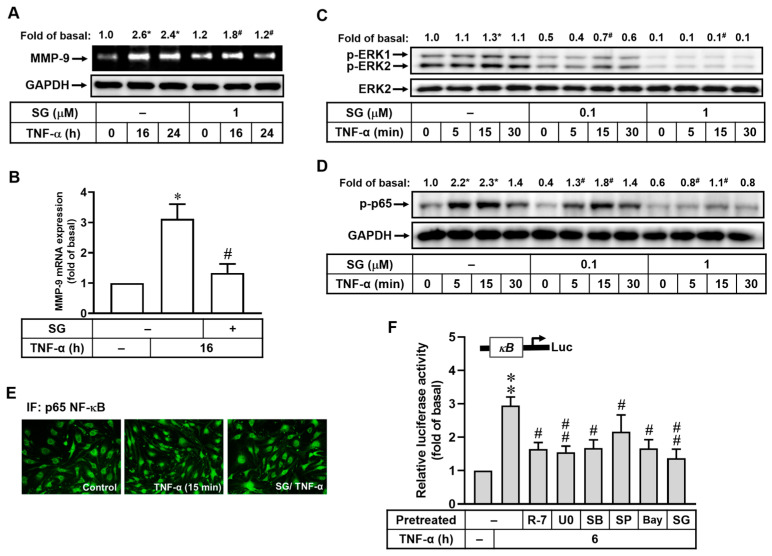
Effects of SG on TNF-α-induced MMP-9 expression in bMECs. (**A**,**B**) After SG pretreatment (1 μM) and TNF-α (30 ng/mL) stimulation in cells for the indicated time intervals, samples were collected and assayed for MMP-9 expression by gelatin zymography with GAPDH protein expression as control by Western blot (**A**) and RT-PCR (**B**). (**C**,**D**) Cells pretreated with SG (0.1 or 1 μM) for 1 h and stimulated with TNF-α (30 ng/mL) were harvested, and the cell lysates were assessed for phosphorylation of MAPKs (p-ERK1/2), phosphorylation of p65 NF-κB (p-p65), ERK2, and GAPDH by Western blot. (**E**) After SG (1 μM) pretreatment for 1 h and TNF-α (30 ng/mL) incubation for 15 min, cells were fixed and labeled with anti-p65 NF-κB antibody and FITC-conjugated secondary antibody and and imaged. (**F**) After transfection with pGL4.32 reporter construct, the cells were pretreated with R-7050 (R-7, 1 μM), U0126 (U0, 1 μM), SB202190 (SB, 30 μM), SP600125 (SP, 10 μM), Bay11-7082 (Bay, 1 μM), and SG (1 μM) for 1 h and then stimulated with TNF-α (30 ng/mL) for 6 h. The relative promoter activity was measured as firefly luciferase activity to that of renilla luciferase activity. Data are expressed as the mean ± SEM (*n* = 3). * *p* < 0.05; ** *p* < 0.01 (compared to that of untreated control). ^#^ *p* < 0.05; ^##^ *p* < 0.01 (compared to TNF-α stimulation only). The image represents one of three individual experiments.

Based on the results, we demonstrated that TNF-α induces MMP-9 expression and thereby disrupts the ZO-1 arranged integrity in bMECs. Therefore, we evaluated the effects of the natural flavanone compound SG on the aforementioned phenomenon. To achieve this objective, cells were pretreated with SG (1 μM) for 1 h and following TNF-α stimulation for the indicated times. In Figure 5A, SG pretreatment significantly blocked TNF-α-induced MMP-9 expression in bMECs. To further analyze the effect on MMP-9 transcription level, SG pretreatment attenuated TNF-α-induced MMP-9 mRNA expression in bMECs (Figure 5B). These results indicated that SG might have brain-protective potential by reducing MMP-9 expression induced by TNF-α in bMECs. Next, we determined the effects of SG on the signaling pathway of TNF-α-induced MMP-9 expression, including ERK1/2, p38, JNK1/2 MAPKs, and NF-κB. As shown in Figure 5C, SG pretreatment (1 μM) markedly reduced TNF-α-stimulated phosphorylation of ERK1/2 both in time- and concentration-dependent manners but not p38 and JNK1/2. These results indicated that SG can inhibit TNF-α-induced MMP-9 expression by attenuating ERK1/2 activation in bMECs.

The effect of SG on TNF-α-stimulated p65 NF-κB phosphorylation, translocation, and transcriptional activity were further explored in bMECs. The results showed that pretreatment with SG (1 μM) significantly blocked TNF-α-stimulated p65 NF-κB phosphorylation in time- and concentration- dependent manners (Figure 5D). Additionally, we also confirmed that pretreatment with SG (1 μM) markedly inhibited TNF-α-stimulated translocation of p65 NF-κB into the nucleus by immunofluorescence stain (Figure 5E). Moreover, previous studies have demonstrated that there is a regulative NF-κB binding site in the MMP-9 promoter region [15]. Thus, we next elucidated whether TNF-α can induce NF-κB transcriptional activity and the effect of SG on this event; a pGL4.32 plasmid with a κB response element was used. As shown in Figure 5F, TNF-α markedly induced the transcriptional activity of κB at 6 h (~3 fold), which was significantly diminished by pre-incubating with R-7050 (R-7, 1 μM), U0126 (U0, 1 μM), SB202190 (SB, 30 μM), SP600125 (SP, 10 μM), or Bay11-7082 (Bay, 1 μM), suggesting that TNF-α induces NF-κB activation via the TNFR/MAPKs-dependent pathway in bMECs. Here, we further investigated the interaction modes between SG and TNFR protein by DS molecular docking. The molecular docking result indicated that SG can interact with the key protein of TNFR (Figure S1). All these data demonstrate that TNFR/MAPKs/NF-κB cascade plays a regulative role in TNF-α-induced MMP-9 expression in bMECs. As expected, pretreatment with SG (1 μM) also significantly repressed the response (Figure 5F), demonstrating that SG may play the role of an inhibitor in TNF-α-induced MMP-9 expression by blocking NF-κB activation in bMECs.

### 2.6. Effects of Sophoraflavanone G on TNF-α-Induced MMP-9-Mediated Disruption of ZO-1 Arranged Integrity in bMECs

**Figure 6:**
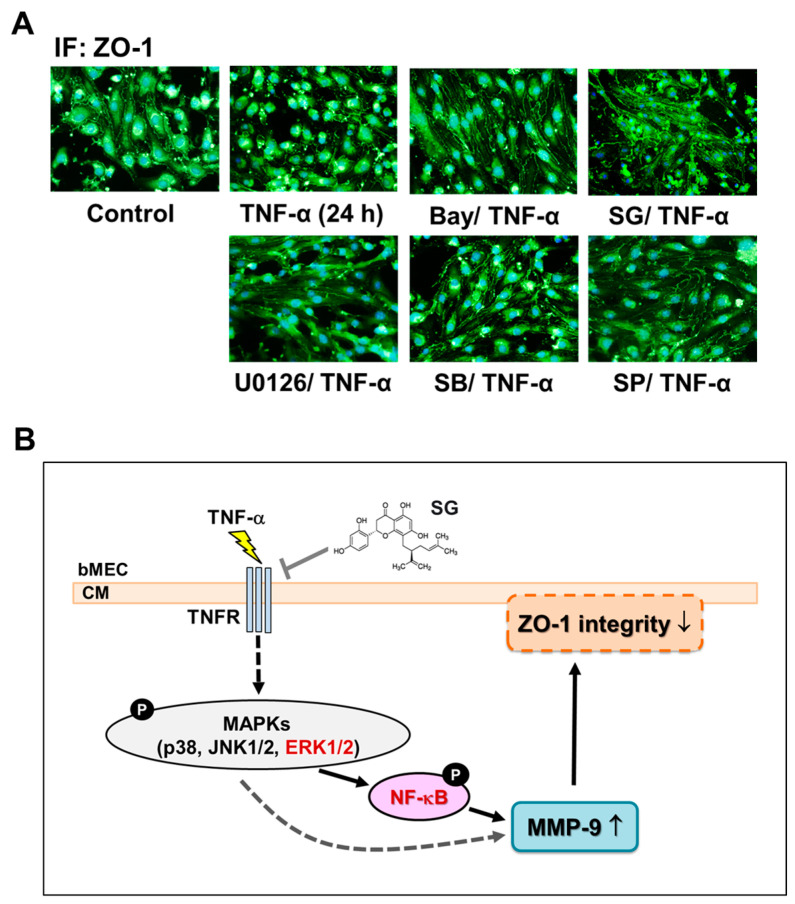
Effects of SG on TNF-α-induced MMP-9-mediated disruption of ZO-1 arranged integrity in bMECs. (**A**) Cells grown on coverslips were pretreated with U0126 (1 μM), SB202190 (SB, 30 μM), SP600125 (SP, 10 μM), Bay11-7082 (Bay, 1 μM), and SG (1 μM) for 1 h and stimulated by TNF-α (30 ng/mL) for 24 h. After fixing and labeling with anti-ZO-1 and fluorescein isothiocyanate (FITC)-conjugated antibody, the immunofluorescence image represents the results from one of three individual experiments. (**B**) Schematic presentation of the brain-protective effects of SG on the disruption of ZO-1 integrity induced by TNF-α-stimulated MMP-9 in bMECs. TNF-α induces MMP-9 expression by MAPKs activation via TNFR-mediated NF-κB-dependent pathway. The upregulated MMP-9 leads to disruption of ZO-1 integrity in bMECs. SG blocks the disruption of ZO-1 integrity by TNF-α-induced MMP-9 via inhibiting TNFR-dependent activation of MAPKs (i.e., ERK1/2, p38, and JNK1/2) and the NF-κB signaling pathway. The solid arrow indicates that its upstream and downstream relationships have been confirmed. The dotted arrow indicates that there are still unknown molecules involved.

Finally, we wanted to see if SG had any influence on the depletion of ZO-1 arranged integrity in bMECs resulting from TNF-α-induced MMP-9. To determine the effect, cells were pretreated with U0126 (1 μM), SB202190 (SB, 30 μM), SP600125 (SP, 10 μM), Bay11-7082 (Bay, 1 μM), or SG (1 μM) and then incubated with TNF-α for 24 h. The results obtained from the analysis of the image data showed that the disruption of ZO-1 arranged integrity induced by TNF-α-stimulated MMP-9 was inhibited by pretreatment of U0126, SB202190, SP600125, or Bay11-7082 (Figure 6A), suggesting that activation of MAPKs (ERK1/2, p38, and JNK1/2) and NF-κB is critical for TNF-α-induced disruption of ZO-1 arranged integrity. Moreover, SG pretreatment can inhibit TNF-α-induced disruption of ZO-1 arranged integrity (Figure 6A, SG/TNF-α). The results suggested that SG blocks TNF-α-induced disruption of ZO-1 arranged integrity via inhibiting the related signaling pathway (i.e., TNFR-ERK1/2-NF-κB cascade) of MMP-9 upregulation in brain microvascular endothelial cells.

## 3. Discussion

MMPs have a significant role in various biological processes across diverse tissues, including major CNS disorders such as stroke, neurodegenerative diseases, and malignant glioma. Among MMPs, MMP-9 expression and activation play a critical role not only in tissue remodeling but also in the pathogenesis of brain diseases [10,11,27], such as being a risk factor in the integrity of the BBB [28]. The BBB disruption is a prominent characteristic observed in CNS illnesses that involve neuroinflammation. Cerebrovascular cells, namely bMECs, play a crucial role in preserving the microenvironment and functional integrity of the BBB [1]. The brain can be protected from BBB disruption, cell death, and enhanced neuroinflammation through the inhibition of MMP activity using pharmacological inhibitors or gene knock-out techniques [28,29]. Some proinflammatory cytokines such as TNF-α and IL-1β have been shown to induce the expression of MMP-9 following brain injury, including BBB damage [30,31]. Our previous data have demonstrated that cytokine IL-1β-induced MMP-9 expression in brain astrocytes may cause astrocytic motility [16]. The findings of these researches indicate that the increase in MMP-9 expression caused by proinflammatory mediators could have a significant impact on brain injury and BBB disruption. These results could provide a potential therapeutic approach for addressing brain inflammatory response even for neurodegenerative conditions. The research from the pharmacological studies and knockout mice has demonstrated that MMP-9 and its upstream signaling pathways may be therapeutic targets in brain injury and inflammatory disorders. In this study, we first demonstrated that TNF-α can induce MMP-9 expression with the detailed underlying mechanisms and their effects in bMECs. Next, we evaluated whether SG, the natural flavanone product, possesses brain-protective effects on TNF-α-induced MMP-9-related events and its underlying mechanism. All these data suggested that in bMECs, SG inhibited TNF-α-induced MMP-9-mediated disruption of ZO-1 arranged integrity through blocking the TNFR-dependent MAPKs (ERK1/2) cascade leading to activation of the NF-κB pathway.

In our study, we first found that TNF-α can upregulate MMP-9 expression even in transcriptional and translation events in bMECs. Next, we demonstrated that TNF-α-mediated upregulation of MMP-9 can affect the main tight junction protein ZO-1 arranged integrity, which is closely associated with the degree of BBB destruction, and it becomes a symbol of the BBB dysfunction [6,32]. Our results further showed that TNF-α-induced disruption of ZO-1 integrity mediated by MMP-9 in bMECs was inhibited by TNFR antagonist R-7050, including expression of mRNA, protein, and disruption of ZO-1 integrity. These data suggest that the TNF/TNFR system plays a critical role in MMP-9-dependent disruption of ZO-1 integrity in BBB breakdown, where the observation is consistent with previous results [7,33]. To explore the protective natural compound for the event, a natural flavanone compound SG was evaluated. The results showed that SG (1 μM) attenuated TNF-α-induced MMP-9 expression in bMECs both in transcriptional and translational levels (Figure 5A,B). Here, we also found that SG can interact with the key protein of TNFR by DS molecular docking (Figure S1). Moreover, pretreatment of SG also affected TNF-α-induced disruption of ZO-1 arranged integrity (Figure 6A), suggesting that SG may have a brain-protective effect by blocking TNF-α-induced events such as BBB dysfunction. These results are the first finding that SG can reduce the disruption of ZO-1 integrity through the MMP-9 pathway in TNF-α-challenged bMECs.

MAPKs act as a pivotal inflammatory event through activation of MAPK-dependent cascades in different cell types [34,35]. In our observation of the signaling of TNF-α-induced MMP-9 expression in bMECs, MAPKs also serve as second messengers to modulate inflammatory responses. In several experimental models with CNS inflammation and injury, the activity of MAPKs is dysregulated [34,35]. Several studies also indicate that proinflammatory factors induced MMP-9 expression in various cells via the MAPKs-dependent pathways [36]. In brain astrocytes, our previous studies have demonstrated that activation of ERK1/2, p38, and JNK1/2 are required for MMP-9 upregulation by proinflammatory cytokines [15,16]. Here, our data showed that the MAPKs participated in MMP-9 expression upon TNF-α stimulation in bMECs via the TNFR-mediated pathway (Figure 3). The findings are similar with the report showing that IL-1β induces MMP-9 expression via ROS-dependent MAPKs in rat brain astrocytes [16]. Moreover, SG markedly inhibited TNF-α-stimulated phosphorylation of ERK1/2 (Figure 5C) but not p38 MAPK and JNK1/2 in these cells. All these results indicated that SG can reduce TNF-α-induced MMP-9 expression through blocking the ERK1/2-dependent cascade in bMECs. The SG data are consistent with the previous reports showing that SG has anti-inflammatory activity by blocking the MAPKs-mediated pathway in LPS-activated mouse macrophages [37] and BV2 microglia [38] and *Streptococcus mutans* surface antigen I/II-challenged RAW 264.7 macrophages [39]. These results suggested that MAPKs (ERK1/2) may also be effective and novel therapeutic targets for CNS-related diseases.

The progressive increase of MAPKs activity during the action of several proinflammatory factors not only causes damage signaling transduction but also modulates the gene expression pattern through functional alterations of transcription factors. The transcription factor NF-κB plays a critical role in the modulation of functional gene expressions in physiological and pathological events, such as inflammation, cell proliferation, and apoptosis [12,40]. The induction of inflammatory mediators, such as MMP-9, in brain cells during brain inflammation is a result of numerous stimuli. This induction occurs through the activation of NF-κB in a MAPKs-mediated way [12]. Our previous study demonstrated that NF-κB contributes to the upregulation of several genes, including MMP-9, by proinflammatory cytokines through a MAPKs-mediated manner in brain astrocytes [12]. These results indicate that NF-κB is critical for upregulation of several inflammatory genes such as MMP-9 in the CNS inflammation [12,41]. Therefore, in the present investigation, we focused on the effects of SG on TNF-α-stimulated NF-κB activation in bMECs. The transcription factor NF-κB participated in TNF-α-induced MMP-9 expression (Figure 4). Moreover, the TNFR-dependent activation of MAPKs (i.e., ERK1/2, p38, and JNK1/2) cascades was involved in TNF-α-stimulated NF-κB activation (Figure 4E). We further demonstrated that TNF-α-stimulated activation of p65/NF-κB, including phosphorylation, nuclear translocation, and transcriptional activity, which were significantly suppressed by SG (Figure 5D,F). These results demonstrated that SG can reduce TNF-α-induced MMP-9 expression via inhibiting NF-κB activation in bMECs. These results are consistent with the idea that SG inhibits the expression and production of inflammatory mediators by blocking the ERK/MAPK-mediated pathway and inhibiting the activation of NF-κB in macrophages [37,39]. Finally, our data showed that increased MMP-9 expression by TNF-α stimulation was via TNFR-mediated MAPKs signals leading to activation of NF-κB, resulting in the disruption of ZO-1 integrity (representing BBB damage) in bMECs (Figure 6A). In this study, we also found that SG can alleviate this MMP-9-mediated event by inhibiting a series of signaling in bMECs, as mentioned in the text. However, there still exists an obstacle in clinical use due to the difficulties in flavonoid administration. Because of low bioavailability, SG needs to be formulated using some smart drug release and/or delivery technologies, like other flavonoid compounds [42,43,44]. Therefore, the optimal delivery system for SG needs to be further verified, especially in the CNS system.

## 4. Materials and Methods

### 4.1. Materials

Dulbecco’s modified Eagle’s medium (DMEM)/F-12 medium, fetal bovine serum (FBS), and TRIzol were from Invitrogen (Carlsbad, CA, USA). The Hybond C membrane and enhanced chemiluminescence (ECL) Western blot detection system were from GE Healthcare Biosciences (Buckinghamshire, UK). The phospho-ERK1/2 (Thr202/Tyr204) (Cat.#4370), phospho-p38 (Thr180/Tyr182) (Cat.#4511), phospho-JNK1/2 (Thr183/Tyr185) (Cat.#4668), and phospho-p65 NF-κB (Ser536) (Cat.#3033) antibodies were from Cell Signaling (Danver, MA, USA). The anti-ZO-1 (Cat.#sc-33725) antibody was from Santa Cruz (Dallas, TX, USA). The anti-glyceraldehyde-3-phosphate dehydrogenase (GAPDH) antibody was from GeneTex (Irvine, CA, USA). The R-7050, U0126, SB202190, SP600125, Bay11-7082, and MMP2/9 inhibitor (2/9i) were from Enzo (Farmingdale, NY, USA). The bicinchoninic acid (BCA) protein assay reagent was from Pierce (Rockford, IL, USA). The tumor necrosis factor-α (TNF-α) was from R&D Systems (Minneapolis, MN, USA). The sophoraflavanone G (SG) was from ChemFaces (Wuhan, China). The enzymes and other chemicals were from Sigma (St. Louis, MO, USA).

### 4.2. Cell Cultures and Treatments

Mouse brain microvascular endothelial cells (bMECs: bEnd.3) were cultured and used throughout this study as described previously [45]. Cells were grown in DMEM/F-12 containing 10% FBS and antibiotics (100 U/mL penicillin G, 100 μg/mL streptomycin, and 250 ng/mL fungizone) at 37 °C in a humidified 5% CO_2_ atmosphere. Confluence cells were released with 0.05% (*w*/*v*) trypsin/0.53 mM EDTA for 5 min at 37 °C. The cell suspension (2 × 10^5^ cells/mL) was plated onto 6-well culture plates (2 mL/well) or 10 cm culture dishes (10 mL/dish) for the measurement of protein or RNA expression, respectively. Culture medium was changed after 24 h and then every 3 days. Experiments were performed with cells from passages 5 to 13. When the inhibitors were used, cells were pretreated with the inhibitor for 1 h before exposure to TNF-α (30 ng/mL). Treatment of bMECs with these inhibitors alone had no significant effect on cell viability determined by an XTT assay.

### 4.3. MMP Gelatin Zymography

Growth-arrested cells were incubated with TNF-α for the indicated time intervals. After treatment, the cultured media were collected and analyzed by gelatin zymography [15]. Gelatinolytic activity was manifested as horizontal white bands on a blue background. Because cleaved MMPs were not reliably detectable, only pro-form zymogens were quantified.

### 4.4. Total RNA Extraction and Reverse Transcription-PCR Analysis

Total RNA was extracted as described previously [45]. The cDNA obtained from 0.5 μg total RNA was used as a template for PCR amplification. Oligonucleotide primers were designed on the basis of Genbank entries for mouse MMP-9 and β-actin. The primers were as follows: *MMP-9*: 5′-GCTGACTACGATAAGGACGGCA-3′ (sense), 5′-TAGTGGTGCAGGCAGAGTAGGA-3′ (antisense); *β-actin*: 5′-AGAGGGAAATCGTGCGTGAC-3′ (sense), 5′-CAATAGTGATGACCTGGCGT-3′ (anti-sense). The amplification was performed in 30 cycles at 55 °C, 30 s; 72 °C, 1 min; 94 °C, 30 s. PCR fragments were analyzed on 2% agarose 1 × TAE gel containing ethidium bromide, and their size was compared with the molecular weight markers. Amplification of β-actin, a relatively invariant internal reference RNA, was performed in parallel, and cDNA amounts were standardized to equivalent β-actin mRNA levels. The images were quantified and analyzed by UN-SCAN-IT gel version 6.1 software (Silk Scientific, Inc., Orem, UT, USA).

### 4.5. Western Blot Analysis

Growth-arrested cells were incubated with TNF-α at 37 °C for the indicated time intervals. The cells were washed with ice-cold PBS, scraped, and collected by centrifugation at 45,000× *g* for 1 h at 4 °C to yield the whole cell extract, as previously described [15]. Samples were analyzed by Western blot, transferred to nitrocellulose membrane, and then incubated overnight using an anti-phospho-ERK1/2, phospho-p38, phospho-JNK1/2, phospho-p65 NF-κB, or GAPDH antibody. Membranes were washed four times with TTBS for 5 min each, incubated with a 1:2000 dilution of anti-rabbit horseradish peroxidase antibody for 1 h. The immunoreactive bands were detected by ECL reagents and captured by a UVP BioSpectrum 500 Imaging System (Upland, CA, USA). The image densitometry analysis was quantified by UN-SCAN-IT gel 6.1 software (Orem, UT, USA).

### 4.6. Immunofluorescence Stain

Growth-arrested cells were treated with 30 ng/mL TNF-α for the indicated time intervals, washed twice with ice-cold PBS, fixed with 4% (*w*/*v*) paraformaldehyde in PBS for 30 min, and then permeabilized with 0.3% Triton X-100 in PBS for 15 min. The stain was performed by incubating with 10% normal goat serum in PBS for 30 min followed by incubating with an anti-p65 NF-κB or ZO-1 antibody (1:200 dilution) for 1 h in PBS with 1% BSA, washing thrice with PBS, incubating for 1 h with a FITC conjugated goat anti-rabbit antibody (1:200 dilution) in PBS with 1% BSA, washing thrice with PBS, and finally mounting with aqueous mounting medium. The images were observed under a fluorescence microscope (Axiovert 200 M, Carl Zeiss, Göttingen, Germany).

### 4.7. Promoter-Luciferase Reporter Gene Assay

The κB binding sites were cloned to the pGL4.32 (luc2P/NF-κB-RE/Hygro) vector containing the luciferase reporter system. All plasmids were prepared by using QIAGEN plasmid DNA preparation kits. These constructs were transfected into bEnd.3 cells by using a Lipofectamine reagent according to the instructions of the manufacturer. The transfection efficiency (~60%) was determined by transfection with enhanced GFP. After incubation with TNF-α, cells were collected and disrupted by sonication in lysis buffer (25 mM Tris, pH 7.8, 2 mM EDTA, 1% Triton X-100, and 10% glycerol). After centrifugation, aliquots of the supernatants were tested for promoter activity using a Dual-Luciferase^®^ Reporter Assay System (Promega, Madison, WI, USA). Firefly luciferase activities were standardized for Renilla luciferase activity.

### 4.8. Statistical Analysis

All data were estimated using the GraphPad Prism Program (GraphPad, San Diego, CA, USA, https://www.graphpad.com/, accessed on 1 December 2023). Quantitative data were analyzed by one-way ANOVA followed by Tukey’s honestly significant difference tests between individual groups. Data were expressed as mean ± SEM. A value of *p* < 0.05 was considered significant.

## 5. Conclusions

Based on our observations and findings, Figure 6B depicts a brain-protective model of SG on TNF-α-mediated disruption of ZO-1 integrity in bMECs. Herein, SG pretreatment markedly inhibits the activation of related signaling molecules in TNF-α-induced MMP-9 expression, including ERK1/2-dependent activation of the NF-κB pathway. These results are the first evidence indicating that SG can inhibit MMP-9-mediated events in bMECs. These findings concerning the natural flavanone compound SG reduces TNF-α-induced ERK1/2-mediated NF-κB and MMP-9-dependent disruption of ZO-1 integrity in bMECs, suggesting that SG may be a promising natural candidate for relieving brain inflammatory events.

## Data Availability

The data that support the findings of this study are available from the authors upon reasonable request.

## Supplementary Materials

The following supporting information can be downloaded at https://www.mdpi.com/article/10.3390/ijms25010283/s1.
