# Supplementary material for: Protective Effects of Sophoraflavanone G by Inhibiting TNF-α-Induced MMP-9-Mediated Events in Brain Microvascular Endothelial Cells
Source: Int J Mol Sci. 2023 Dec 24;25(1):283. doi: 10.3390/ijms25010283 (PMC10779338; doi:10.3390/ijms25010283)
Supplement: Supplementary file 1 [file ijms-25-00283-s001.zip › ijms-2779143-supplementary.pdf]

Supplementary information

Method

Molecular Docking

The molecular docking software used here was Discovery Studio 2021 Client. The preparation process of the target protein as follows: The crystal structure of the target proteins of TNFR (PDB ID: 1TNR) was downloaded from the PROTEIN DATA BANK (https://www.rcsb.org/). The grid boxes after identifying the binding site of the protein and its residues were created. We visualized and analyzed the ligand-protein complexes and used the LibDock scores to predict binding affinities between SG and TNFR protein.

Result

Here, we further investigated the interaction modes between SG and TNF receptor (TNFR) protein by DS molecular docking. The molecular docking data indicated that SG can interact with the key protein of TNFR (Figure S1). Thus, we suggested that SG can inhibit TNF-α-induced MMP-9-mediated disruption of ZO-1 integrity in bMECs via blocking TNFR-dependent activation of MAPKs (i.e., ERK1/2, p38, and JNK1/2) and NF-κB signaling pathway.

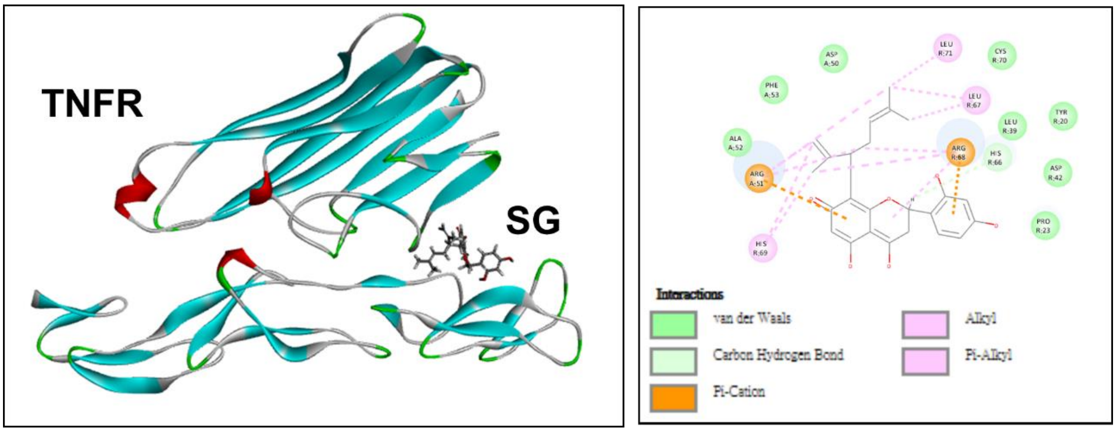

Figure S1. Molecular docking about SG and the TNFR protein. Two-dimensional binding modes and three-dimensional H-binding modes showing the interactions between SG and TNFR protein.
